# The Diagnostic Value of Mean Apparent Propagator‐MRI in Dysthyroid Optic Neuropathy: A Quantitative Analysis of the Entire Visual Pathway

**DOI:** 10.1002/cns.70793

**Published:** 2026-02-11

**Authors:** Mengsha Zou, Dide Wu, Yanglei Cheng, Haodong Qin, Zhiyun Yang, Shubin Hong, Hongzhang Zhu, Cui Yan

**Affiliations:** ^1^ Department of Radiology The First Affiliated Hospital of Sun Yat‐Sen University Guangzhou China; ^2^ Department of Endocrinology The First Affiliated Hospital of Sun Yat‐Sen University Guangzhou China; ^3^ MR Research Collaboration Siemens Healthineers Guangzhou China

**Keywords:** dysthyroid optic neuropathy, intracranial visual pathway, mean apparent propagator MRI, orbital

## Abstract

**Objectives:**

This study aimed to apply mean apparent propagator‐MRI (MAP‐MRI) to the entire visual pathway extending from the orbital to the intracranial visual pathway, to evaluate the model performance in diagnosing dysthyroid optic neuropathy (DON).

**Methods:**

57 thyroid‐associated ophthalmopathy (TAO) patients including 30 with DON (55 eyes) and 27 without DON (54 eyes) were collected in this study. Orbital MAP‐MRI parameters of the optic nerve (ON) and intracranial visual pathway MAP‐MRI parameters of the optic tract (OT), optic radiation (OR), and Brodmann areas (BAs) 17, 18, and 19 were measured and compared. Diagnostic models were constructed based on parameters with significant differences, and the diagnostic performance of models was evaluated and compared using receiver operating characteristic curve analysis and the DeLong test.

**Results:**

The DON group showed significantly higher values of q‐space inverse variance (QIV) and mean squared displacement (MSD), and lower values of non‐Gaussianity (NG), radial non‐Gaussianity (NGRad), return‐to‐axis probability (RTAP), return‐to‐origin probability (RTOP), and return‐to‐plane probability (RTPP) (*p* < 0.05) of the ON than the non‐DON group. As for the intracranial visual pathway, NGRad values of OT and QIV, MSD values of BA17 were all higher in the DON group (*p* < 0.05). The model combining orbital and intracranial visual pathway MAP‐MRI parameters achieved the best diagnostic performance (AUC = 0.873), which showed a significant improvement over the simple orbital or intracranial visual pathway model.

**Conclusion:**

Orbital and intracranial visual pathway MAP‐MRI both achieved certain efficacy in diagnosing DON. The diagnostic model combining orbital and intracranial MAP‐MRI parameters could significantly optimize diagnostic efficiency.

## Graphical abstract

The diagnostic model combining orbital and intracranial MAP‐MRI parameters could significantly optimize diagnostic efficiency of dysthyroid optic neuropathy. Comprehensive MAP‐MRI parameters analysis of the entire visual pathway enables a deeper understanding of disease involvement, provides novel insights into the early accurate diagnosis and therapeutic management of DON patients.

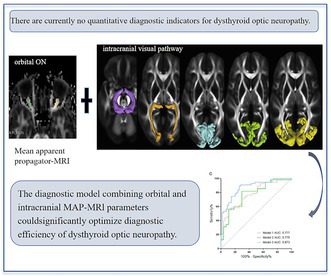

**Abbreviations**
AUC: area under the curve
BA: Brodmann area
CAS: clinical activity score
dMRI: diffusion magnetic resonance imaging
DON: dysthyroid optic neuropathy
DSI: diffusion spectral imaging
FT3: free triiodothyronine
FT4: free thyroxine
HC: healthy controls
MAP‐MRI: mean apparent propagator magnetic resonance imaging
MSD: mean squared displacement
NG: non‐Gaussianity
NGAx: axial non‐Gaussianity
NGRad: radial non‐Gaussianity
ON: optic nerve
OR: optic radiation
OT: optic tract
PDF: probability density function
QIV: q‐space inverse variance
ROC: receiver operating characteristic
RTAP: return‐to‐axis probability
RTOP: return‐to‐origin probability
RTPP: return‐to‐plane probability
TAO: thyroid‐associated ophthalmopathy
TG: thyroglobulin
TGAb: thyroglobulin antibody
TPOAb: thyroid peroxidase antibody
TRAb: thyrotropin receptor antibody
TSH: thyroid stimulating hormone

## 1 Introduction

Dysthyroid optic neuropathy (DON) is a severe vision‐threatening complication of thyroid‐associated ophthalmopathy (TAO), which can lead to permanent visual loss in critical cases [1]. DON exhibits an insidious onset and rapid progression, affecting approximately 4%–8% of TAO patients [2]. The diagnosis of DON mainly relies on clinical signs and symptoms related to visual impairment, such as declined visual acuity, visual field defects, and color vision abnormalities [3]. However, these clinical manifestations lack sufficient sensitivity and specificity due to their similarity to TAO patients without DON, often resulting in early misdiagnosis or underdetection [4]. Therefore, it is of paramount significance to explore early quantitative diagnostic indicators for DON, to facilitate timely detection, promote optic nerve protection, and prevent irreversible vision loss.

The orbital apex crowding syndrome is widely considered the pathophysiology of DON, which leads to compression, stretching, and ischemia of the optic nerve [5]. Thus, most research has centered on orbital structural anomalies, including enlargement of extraocular muscles, expansion of orbital fat, stretching of the optic nerve, and alteratives in quantitative magnetic resonance imaging (MRI) parameters, all of which point to impairment of the anterior visual pathway in DON patients [6, 7, 8, 9, 10]. However, a survey reported that DON can occur in TAO patients without apical muscle crowding, and in the absence of significant extraocular muscle enlargement, suggesting that other factors may contribute to visual loss [2]. Other than orbital MRI, studies have also found significant microstructural changes of the intracranial segment of the visual pathway, including the optic chiasma, optic tract (OT), and optic radiation (OR), in DON patients using diffusion imaging combined with fiber tractography [4, 11]. However, these studies did not investigate microstructural changes in the visual cortex, the region responsible for receiving and processing visual information, which could further contribute to the diagnosis of DON [4, 11, 12]. Therefore, exploring indicators that cover the entire visual pathway from the orbital to the intracranial visual pathway might be more valuable for the exploration of potential pathological mechanisms and the diagnosis of DON. Furthermore, the use of standardized registration and template‐based region of interest (ROI) analysis helps to enhance the objectivity of brain diffusion measurement.

In recent years, diffusion magnetic resonance imaging (dMRI) has been increasingly utilized to investigate microstructural alterations in DON patients [4, 6, 11, 13, 14, 15]. Conventional dMRI techniques are constrained by model assumptions that inadequately characterize the non‐Gaussian distribution of water molecules in complex biological tissues. To address this limitation, mean apparent propagator‐MRI (MAP‐MRI) has been developed as a novel computational framework for diffusion spectral imaging (DSI) [16]. MAP‐MRI models water diffusion without prior assumptions about tissue microstructure, thereby capturing subtler pathological changes in heterogeneous environments. Based on q‐space sampling, MAP‐MRI quantifies the probability density function (PDF) of spin displacement to evaluate water diffusion profiles, characterizing non‐Gaussian diffusion properties to provide more accurate metrics for diffusion anisotropy and microstructural complexity [17, 18]. This technique has demonstrated clinical utility in assessing neurological and oncological conditions such as Parkinson's disease, epilepsy, glioma, and meningioma, validating its sensitivity to microscopic neural tissue changes [19, 20, 21, 22].

Therefore, we aimed to apply this novel method, MAP‐MRI, to the entire visual pathway extending from orbital to intracranial visual pathway to establish a comprehensive and clinically practical diagnostic model for DON. We hypothesized that this combined model would significantly improve the diagnostic performance than models separately based on the orbital or intracranial visual pathway.

## 2 Materials and Methods

This retrospective study was approved by the ethics review committee of our hospital (NO: [2019]061), and each participant provided written informed consent.

### 2.1 Patients

A cohort of 57 TAO patients (female/male: 21/36; mean age 49.05 ± 10.05 years) diagnosed according to European Group on Graves' Orbitopathy (EUGOGO) criteria [23] and 30 age‐ and sex‐matched healthy controls (HC, female/male: 15/15; mean age 49.14 ± 7.95 years) were enrolled from July 2019 to December 2021. Among the TAO group, 30 of the patients with DON were defined as the DON group (unilateral/bilateral = 5/25, 55 eyes), and the other 27 patients without DON were defined as the non‐DON group (54 eyes). There were no significant differences among the DON, non‐DON, and HC groups in age and sex. The diagnosis of DON was made according to the presence of at least any 2 of the following clinical manifestations: (a) the deterioration of visual acuity (VA) < 1.0, (b) loss of color vision, (c) optic disc swelling, and (d) relative afferent pupillary defect [24]. TAO patients who were diagnosed with DON by both blinded ophthalmologists were included in the DON group. Besides, there were some exclusion criteria applied in all participants as follows: (a) local eye disorders due to other diseases such as amblyopia, strabismus, cataract, or eye surgery; (b) psychiatric or neurological disorders such as head injury, bipolar disorder, or schizophrenia; (c) contraindications to MRI examination.

### 2.2 Clinical Assessment

Demographic data including age, sex, and disease duration were collected in our study. Disease duration was defined as the time interval from the onset of ophthalmic symptoms to the performance of MRI. The modified 7‐point clinical activity score (CAS) was used to assess the disease activity. In addition, the clinical parameters involving the free triiodothyronine (FT3), free thyroxine (FT4), thyroid stimulating hormone (TSH), thyrotropin receptor antibody (TRAb), thyroglobulin (TG), thyroglobulin antibody (TG‐Ab), and thyroid peroxidase antibody (TPOAb) levels were measured.

### 2.3 Image Acquisition and Processing

All patients underwent MRI scans via a 3.0 T MR scanner (MAGNETOM Prisma, Siemens Healthineers, Erlangen, Germany) with a 64‐channel head coil. Patients lay supine in the examination bed with foam padding fixing the head to minimize head movement.

Diffusion Spectrum Imaging (DSI) data were acquired using a spin‐echo planar imaging sequence with a half coverage Cartesian q‐space grid scheme and the parameters were as follows: a field of view (FOV) of 220 mm × 220 mm; a repetition time/echo time (TR/TE) of 3700 ms/72 ms; voxel size of 2 × 2 × 2 mm^3^; in‐plane acceleration factor of 2, slice acceleration factor of 2; and a total acquisition time of 9 min. In total, 13 *b*‐values (0, 0.1, 300, 350, 650, 950, 1000, 1350, 1650, 1700, 2000, 2700 and 3000 s/mm^2^) were applied, with corresponding diffusion‐encoding directions of 1, 1, 1, 5, 12, 3, 5, 6, 13, 11, 24, 12 and 6, respectively.

The DSI datasets were processed by NeuDiLab software developed in‐house with Python, which is based on an open‐resource tool DIPY (Diffusion Imaging in Python). The dataset was first corrected for eddy current distortion and simple head motion in reference to the b0 images. Then, the fractional anisotropy (FA) and MAP‐MRI parameter maps were extracted including the non‐Gaussianity (NG), non‐Gaussianity axial (NGAx), non‐Gaussianity vertical (NGRad), q‐space inverse variance (QIV), mean squared displacement (MSD), return to the origin probability (RTOP), return to the axis probability (RTAP), and return to the plane probability (RTPP).

### 2.4 Evaluation of Orbital MAP‐MRI Parameters

Two blinded radiologists with 6 and 4 years of head and neck radiology experience independently analyzed the orbital MAP‐MRI for each eye unit.

**FIGURE 1:**
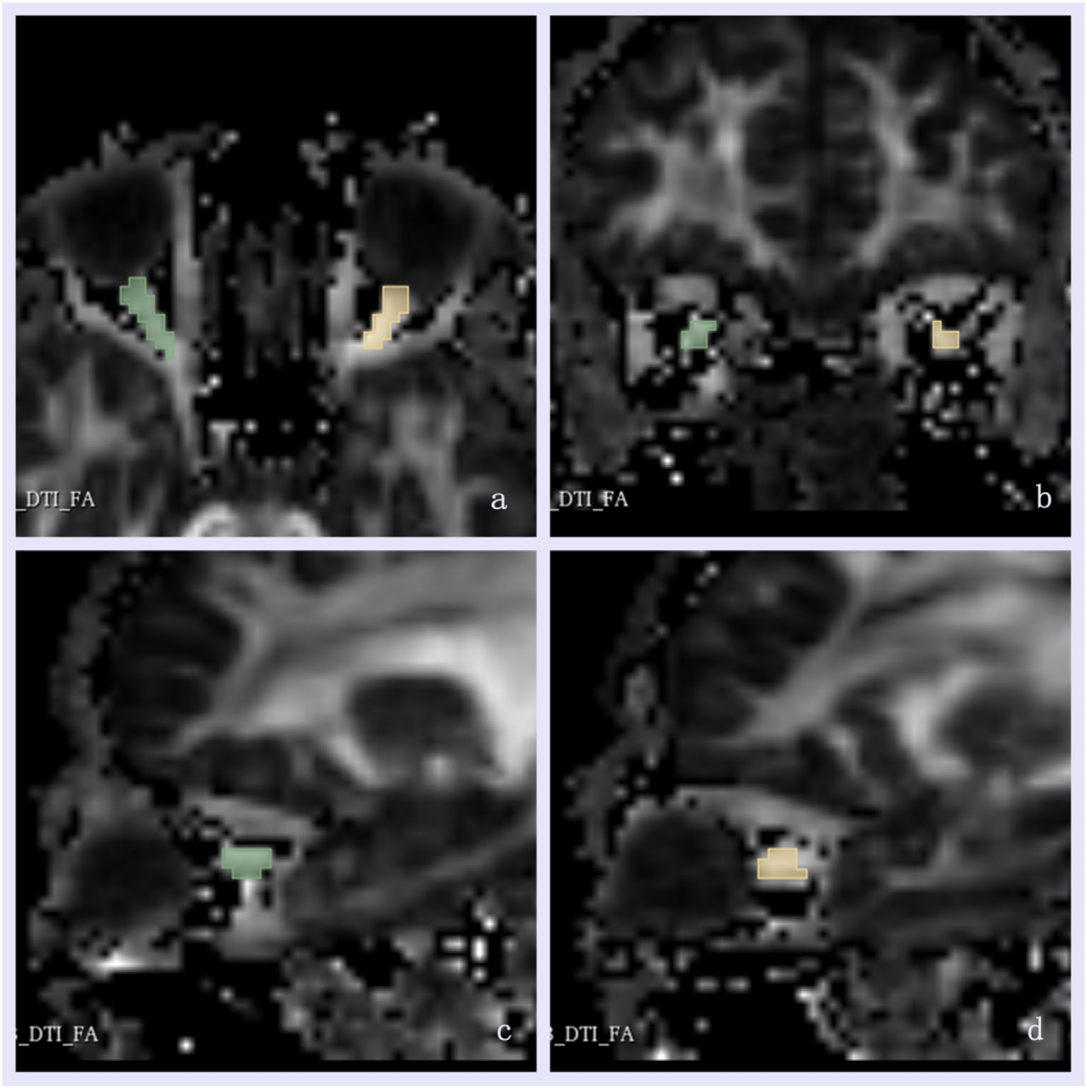
The methods for delineating the optic nerve on the fractional anisotropy diffusion image. The regions of bilateral optic nerve were showed respectively in axial (a), coronal (b), and sagittal planes (c, d), with green indicating the right optic nerve and yellow indicating the left optic nerve.

The open‐resource tool 3D slicer (https://www.slicer.org/) was used to delineate the volume of interest (VOI). For measuring diffusion parameters, VOI was manually drawn around the ON on the FA diffusion image (Figure 1a–d). Then the mask of VOI was applied to all MAP‐MRI parameter maps to extract the orbital MAP‐MRI parameter values within the VOI.

### 2.5 Evaluation of Intracranial Visual Pathway MAP‐MRI Parameters

**FIGURE 2:**
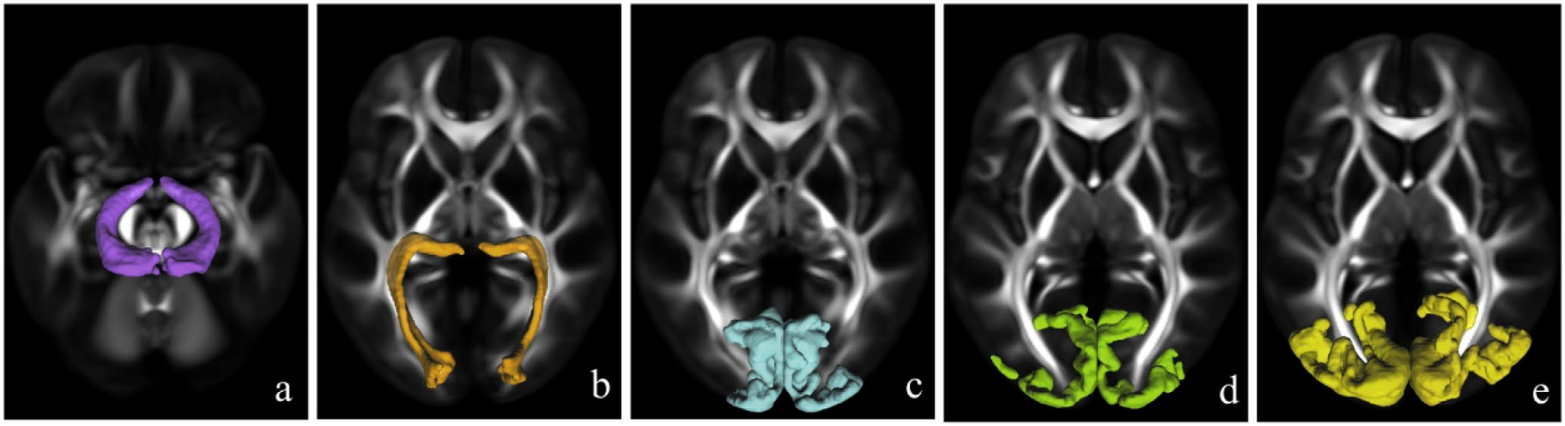
Template‐based masks of the intracranial visual pathway for MAP‐MRI parameter extraction. The mask of OT and OR derived from the HCP‐1065 diffusion template (a, b). The masks of BA 17, 18, and 19 were respectively extracted from the Brodmann area atlas (c–e). BA, Brodmann area; HCP, Human Connectome Project; MAP‐MRI, mean apparent propagator‐magnetic resonance imaging; OT, optic tract; OR, optic radiation.

DSI data were preprocessed using the FMRIB Software Library (FSL, version 5.0, http://www.fmrib.ox.ac.uk/fsl). Subsequent to preprocessing, the MAP‐MRI maps for all participants were spatially normalized to the Montreal Neurological Institute (MNI) standard space using FSL's FMRIB Non‐linear Registration Tool (FNIRT). The standardized masks of the OT, OR based on the Human Connectome Project‐1065 diffusion template, and visual cortex areas BA17, BA18, and BA19 based on the Brodmann area atlas were then applied using DPABI software (http://rfmri.org/dpabi) to extract the corresponding regional MAP‐MRI parameter values (Figure 2a–e). Given the partial decussation at the optic chiasm, which causes unilateral ON lesions to affect the bilateral retrochiasmatic visual pathway, all MAP‐MRI parameter values for OT, OR, BA17, BA18, and BA19 were averaged across both cerebral hemispheres.

### 2.6 Statistical Analysis

Statistical analysis in this study was performed in the SPSS software package (version 23.0; IBM). Continuous variables were expressed as mean ± standard deviation or the median with an interquartile range (25%, 75%) depending on a normal distribution. For data that follow a normal distribution, one‐way ANOVA was used for comparisons among the three groups, followed by independent‐samples *t*‐tests for post hoc pairwise comparisons. For data not conforming to a normal distribution, Kruskal–Wallis tests were applied for group comparisons, with Mann–Whitney *U* tests used for post hoc analysis. The categorical variables were evaluated using the chi‐square test. Group comparison analysis was used for the initial MAP‐MRI parameters selection. These significant altered orbital/visual pathway MAP‐MRI parameters between DON and non‐DON groups were selected as the initial parameters of orbital−/visual pathway‐MAP model for discriminating the DON group from the non‐DON group. Subsequently, a backward‐stepwise multivariate logistic regression analysis was performed with the threshold of *p* < 0.05 to determine the optimal combination of parameters for discriminating the DON group from the non‐DON group. Furthermore, receiver operating characteristic (ROC) curves were drawn, with the area under the curve (AUC), sensitivity and specificity to evaluate the diagnostic performance of the identified significant imaging parameters and combinations. The goodness‐of‐fit of the logistic regression model was evaluated using the Hosmer‐Lemeshow test. Diagnostic performance comparisons were evaluated using the DeLong test [25]. Relationships between MAP‐MRI parameters and clinical variables were assessed via Pearson's or Spearman's correlation analysis, as appropriate for data distribution. Interobserver reproducibility of orbital MAP‐MRI measurements was quantified using intraclass correlation coefficients (ICCs) with the entire cohort. For all of these analyses, Bonferroni correction was performed, using the corrected *p* value. A two‐sided *p* value of < 0.05 was considered significant.

## 3 Results

### 3.1 Demographic and Clinical Information

The demographic characteristics including age and sex were matched among the TAO patients with and without DON, and HC. There were no significant differences in clinical characteristics, including TAO duration, FT3, FT4, TSH, TRAb, TG, TGAb and TPOAb (all *p* > 0.05), between the DON group and non‐DON group. CAS differed significantly between the two groups (all *p* < 0.05) (Table 1).

**TABLE 1:** Demographic and clinical data of the 57 TAO patients and healthy controls.

	DON *N* = 30, 55 eyes	Non‐DON *N* = 27, 54 eyes	HC *N* = 30	*p*
Age (year)	49.87 ± 11.62	48.15 ± 8.10	49.14 ± 7.95	0.785
Sex (male/female)	19/11	17/10	15/15	0.500
TAO duration (year)	1 (0.75, 2)	1 (0.775, 3)	—	0.563
CAS	4 (3, 5)	3 (2, 4)	—	< 0.001^a^
FT3 (pmol/L)	5.50 (4.70, 6.00)	5.30 (4.70, 5.55)	—	0.831
FT4 (pmol/L)	10.30 (9.10, 12.50)	11.70 (9.95, 13.85)	—	0.234
TSH (uIU/ml)	0.68 (0.01, 1.98)	0.43 (0.01, 1.69)	—	0.554
TRAb (IU/L)	8.23 (1.64, 24.29)	6.01 (3.60, 13.88)	—	0.523
TG (ng/ml)	37.36 (5.35, 154.45)	30.65 (1.88, 155.75)	—	0.579
TGAb (IU/mL)	0.006 (0, 0.1)	0.15 (0, 39.29)	—	0.106
TPOAb (IU/mL)	0.50 (0.20, 20.20)	83.55 (0.45, 382.32)	—	0.059

*Note:* The numeric data are reported as the mean ± standard deviation or the median with an interquartile range (25%, 75%).

Abbreviations: CAS, clinical activity score; DON, dysthyroid optic neuropathy; FT3, free triiodothyronine; FT4, free thyroxine; HC, healthy controls; TAO, thyroid‐associated ophthalmopathy; TG, thyroglobulin; TGAb, thyroglobulin antibody; TPOAb, thyroid peroxidase antibody; TRAb, thyrotropin receptor antibody; TSH, thyroid stimulating hormone.

^a^ Statistical significance is indicated by *p* < 0.05.

### 3.2 Orbital MAP‐MRI Parameters Between Groups

**FIGURE 3:**
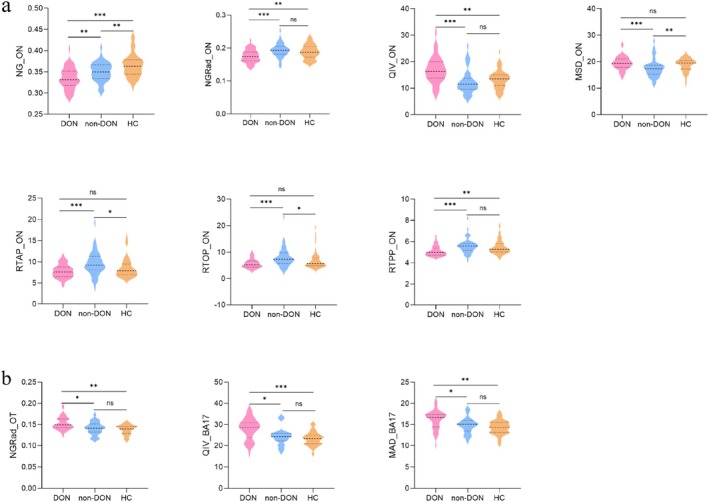
Violin graphs showing the significant quantitative parameters from orbital MAP‐MRI (a) and intracranial visual pathway MAP‐MRI (b) between the DON and non‐DON groups. The statistically significant level, ***p ≤ 0.001; **p < 0.01; *p < 0.05; ns, Not significant. BA, Brodmann area; DON, dysthyroid optic neuropathy; MAP‐MRI, mean apparent propagator‐magnetic resonance imaging; MSD, mean squared displacement; NG, non‐Gaussianity; NGRad, radial non‐Gaussianity; ON, optic nerve; OT, optic tract; QIV, q‐space inverse variance; RTAP, return‐to‐axis probability; RTOP, return‐to‐origin probability; RTPP, return‐to‐plane probability.

Excellent interobserver reproducibility was obtained for all the orbital MAP‐MRI measurements (ICCs, 0.816–0.912). Among the three groups, all MAP‐MRI parameters of ON were significantly different with *p* < 0.001 (Table S1). Compared with the non‐DON group, the DON group showed significantly higher values of QIV and MSD (*p* ≤ 0.001), and lower values of NG (*p* = 0.005), NGRad, RTAP, RTOP, and RTPP (all *p* < 0.001) of the ON. Detailed results between the DON and non‐DON group were presented in Table 2 and Figure 3a.

**TABLE 2:** Significantly altered MAP‐MRI parameters of orbital optic nerve and intracranial visual pathway between the DON and non‐DON groups.

Parameters	DON	Non‐DON	*p*
Orbital‐MAP			
NG_ON	0.34 ± 0.03	0.35 ± 0.02	0.005
NGRad_ON	0.176 ± 0.019	0.193 ± 0.021	< 0.001
QIV_ON	16.33 (13.83, 19.56)	11.57 (9.67, 13.72)	0.001
MSD_ON	19.32 (17.89, 20.86)	17.30 (15.34, 18.66)	< 0.001
RTAP_ON	7.64 (6.62, 8.74)	9.24 (8.12, 11.28)	< 0.001
RTOP_ON	5.25 (4.43, 6.57)	7.36 (5.77, 9.72)	< 0.001
RTPP_ON	4.98 (4.75, 5.35)	5.58 (5.18, 5.83)	< 0.001
Intracranial visual pathway‐MAP			
NGRad_OT	0.15 ± 0.02	0.11 ± 0.02	0.028
QIV_BA17	27.79 ± 5.28	24.31 ± 4.12	0.009
MSD_BA17	16.10 ± 2.31	14.80 ± 1.87	0.045

*Note:* The numeric data are reported as the mean ± standard deviation or the median with an interquartile range (25%, 75%).

Abbreviations: BA, Brodmann area; DON, dysthyroid optic neuropathy; MAP, mean apparent propagator; MSD, mean squared displacement; NG, non‐Gaussianity; NGRad, radial non‐Gaussianity; ON, optic nerve; OT, optic tract; QIV, q‐space inverse variance; RTAP, return‐to‐axis probability; RTOP, return‐to‐origin probability; RTPP, return‐to‐plane probability.

**FIGURE 4:**
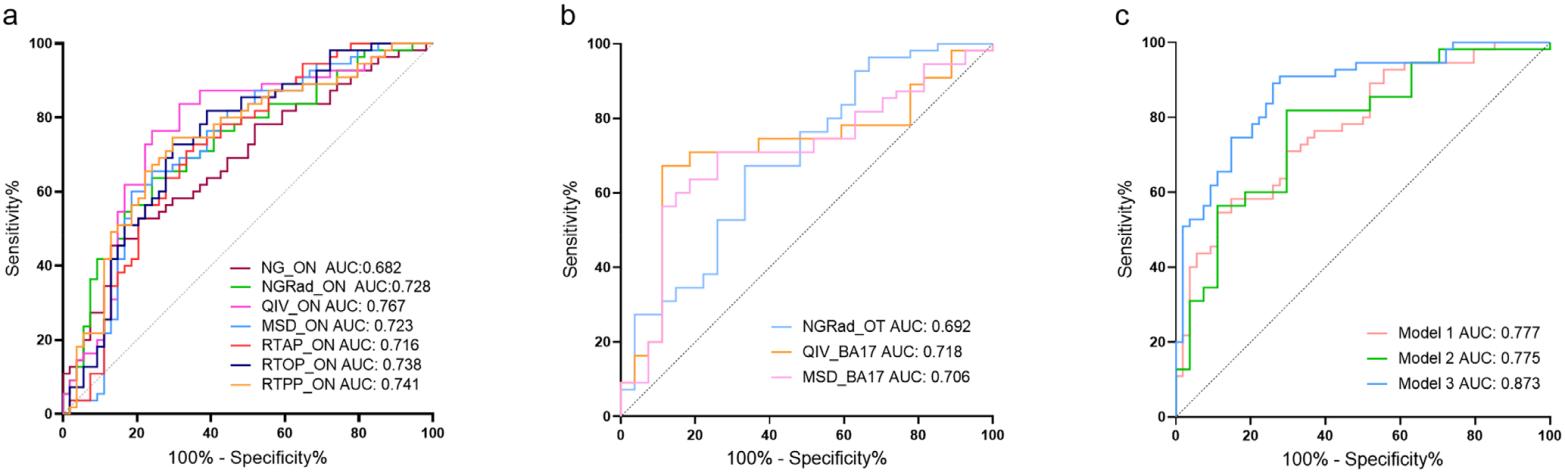
Receiver operating characteristic curves of significant orbital MAP‐MRI based parameters (a), intracranial visual pathway MAP‐MRI based parameters (b), and the diagnostic combinations (c) for diagnosing dysthyroid optic neuropathy, respectively. BA, Brodmann area; MAP‐MRI, mean apparent propagator‐magnetic resonance imaging; MSD, mean squared displacement; NG, non‐gaussianity; NGRad, radial non‐gaussianity; ON, optic nerve; OT, optic tract; QIV, q‐space inverse variance; RTAP, return‐to‐axis probability; RTOP, return‐to‐origin probability; RTPP, return‐to‐plane probability.

The ROC analysis showed that the QIV values of the ON achieved the highest AUC in diagnosing DON (AUC = 0.767, 95% confidence interval [CI]: 0.676–0.843). After the multivariate logistic regression analysis, only three parameters were retained in the final orbital‐MAP model (model 1), including the QIV values, MSD values (AUC = 0.723, 95% CI: 0.629–0.805), and RTOP values (AUC = 0.738, 95% CI: 0.645–0.817) of the ON (Table 3 and Figure 4a).

**TABLE 3:** Diagnostic performances of the single significant image parameters of the orbital‐MAP model and intracranial visual pathway‐MAP model.

	AUC	Cut off	Sensitivity	Specificity	*p*	*p* for model 3
Orbital‐MAP						
NG_ON	0.682 (0.586–0.768)	≤ 0.328	45.45	87.04	0.195	0.207
NGRad_ON	0.728 (0.634–0.808)	≤ 0.182	63.64	75.93	0.355	0.612
QIV_ON	0.767 (0.676–0.843)	> 13.723	76.36	75.93	0.016^a^	0.039^a^
MSD_ON	0.723 (0.629‐0.805)	> 18.913	60.00	81.48	0.019^a^	0.036^a^
RTAP_ON	0.716 (0.622‐0.798)	≤ 8.483	70.91	66.67	0.381	0.238
RTOP_ON	0.738 (0.645–0.817)	≤ 6.420	72.73	70.37	0.027^a^	0.023^a^
RTPP_ON	0.741 (0.648‐0.820)	≤ 5.295	74.55	70.37	0.918	0.943
Intracranial visual pathway‐MAP						
NGRad_OT	0.692 (0.597–0.777)	> 0.145	67.27	66.67	0.007^a^	0.008^a^
QIV_BA17	0.718 (0.624‐0.800)	> 26.516	67.27	88.89	0.003^a^	0.002^a^
MSD_BA17	0.706 (0.611–0.789)	> 16.476	56.36	88.89	0.123	0.231

Abbreviations: AUC, area under the curve; BA, Brodmann area; MAP, mean apparent propagator; MSD, mean squared displacement; NG, non‐Gaussianity; NGAx, axial non‐Gaussianity; NGRad, radial non‐Gaussianity; ON, optic nerve; OR, optic radiation; OT, optic tract; QIV, q‐space inverse variance; RTAP, return‐to‐axis probability; RTOP, return‐to‐origin probability; RTPP, return‐to‐plane probability.

^a^ Statistical significance is indicated by *p* < 0.05 when significant parameters were identified from the stepwise backward LR selection method.

### 3.3 Intracranial Visual Pathway MAP‐MRI Parameters Between Groups

A total of 17 MAP‐MRI parameters, which covered all 5 regions of the intracranial visual pathway, showed significant differences among the three groups with *p* < 0.05 (Table S1). Furthermore, The NGRad values of the OT (*p* = 0.028), QIV (*p* = 0.009) and MSD (*p* = 0.045) values of the BA17 were all higher in the DON group than non‐DON group (Table 2 and Figure 3b).

The highest AUC was achieved by the QIV values of the BA17 (AUC = 0.718, 95% CI: 0.624–0.800) among the three visual pathway MAP‐MRI parameters. Finally, the MSD values of the BA17 (AUC = 0.706, 95% CI: 0.611–0.789) were estimated, and the NGRad values of the OT (AUC = 0.692, 95% CI: 0.597–0.777) and the QIV values of the BA17 were retained in the final intracranial visual pathway‐MAP model (model 2) (Table 3 and Figure 4b).

### 3.4 Diagnostic Combinations and Comparison of Their Performances

All the significant altered seven orbital and three intracranial visual pathway MAP‐MRI parameters between the DON and non‐DON group were placed into the multivariate logistic regression analysis. A diagnostic combination involving the QIV, MSD, RTOP values of the ON, the NGRad values of the OT and the QIV values of the BA17 (all *p* < 0.05) were constructed (model 3).

ROC analysis indicated that the combined model 3 achieved the optimal diagnostic performance with the AUC of 0.873 (95% CI: 0.795–0.929), and optimal diagnostic sensitivity of 89.09%, followed by model 1 (AUC = 0.777, 95% CI: 0.687–0.851) and then model 2 (AUC = 0.775, 95% CI: 0.685–0.850). A non‐significant Hosmer‐Lemeshow statistic (*p* = 0.251) supported the adequacy of the logistic regression model. Significant differences were found in the diagnostic performance between model 3 and model 1 (*p* = 0.008), as well as model 3 and model 2 (*p* = 0.013). However, there was no significant difference between model 1 and model 2 (*p* = 0.979) (Table 4 and Figure 4c).

**TABLE 4:** Diagnostic performances of the combined models.

Model	AUC	Sensitivity	Specificity	*p*
Model 1: Orbital‐MAP				vs. model 2
QIV_ON+MSD_ON+RTOP_ON	0.777 (0.687–0.851)	54.55	88.89	0.979
Model 2: Intracranial visual pathway‐MAP				vs. model 3
NGRad_OT + QIV_BA17	0.775 (0.685–0.850)	81.82	70.37	0.013^a^
Model 3				vs. model 1
Conbined model 1 + 2	0.873 (0.795–0.929)	89.09	74.07	0.008^a^

Abbreviations: AUC, area under the curve; MSD, mean squared displacement; NGRad, radial non‐Gaussianity; ON, optic nerve; OT, optic tract; QIV, q‐space inverse variance; RTOP, return‐to‐origin probability.

^a^ Statistical significance is indicated by *p* < 0.05 when comparisons of diagnostic performances between models 1 and 2, models 2 and 3, and models 3 and 1 were done respectively.

### 3.5 Relationships Between Image Measurements and Clinical Variables

**FIGURE 5:**
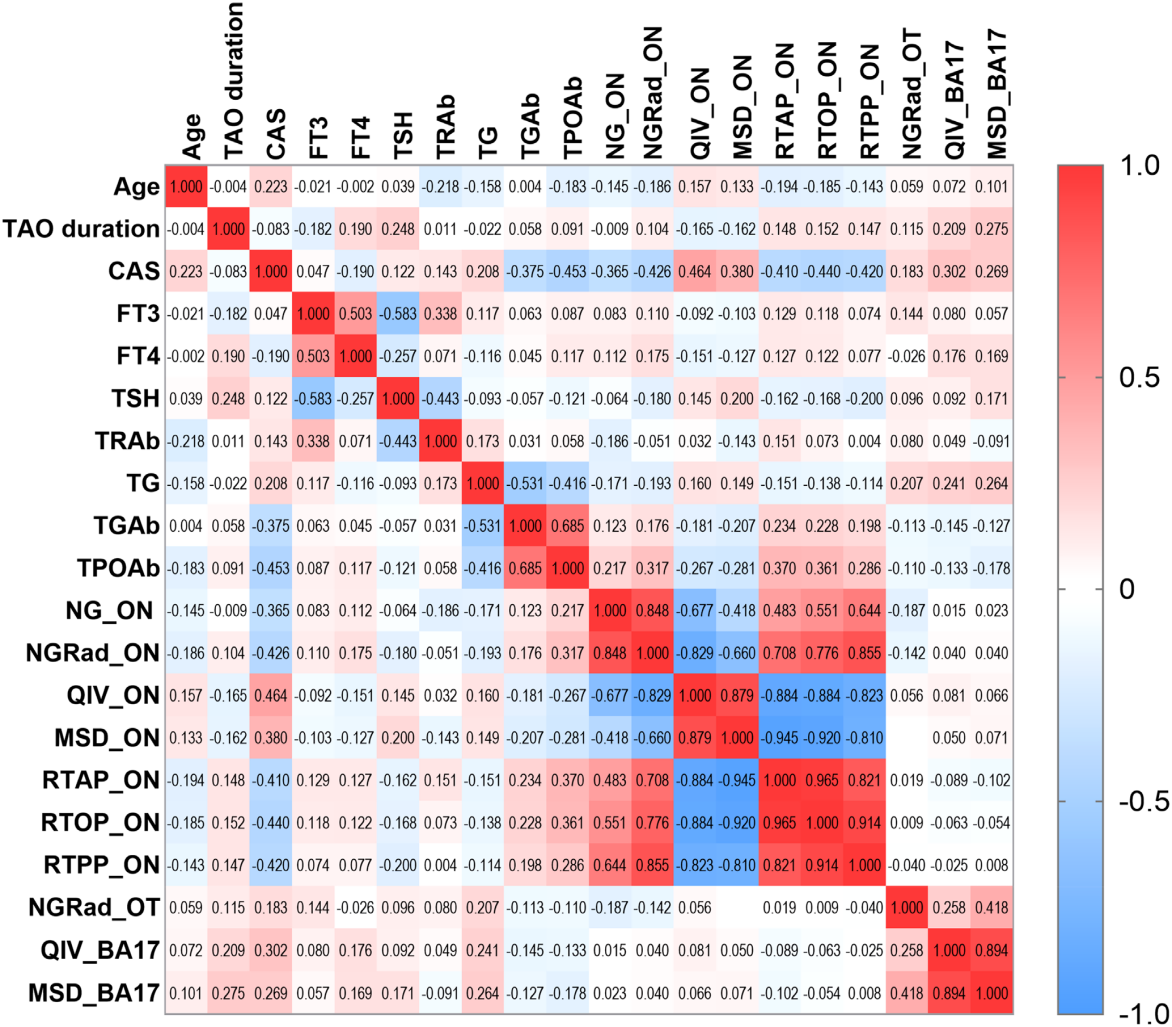
Correlation matrix depicting relationships between image parameters and clinical factors, where each cell represents the correlation coefficient. Red represents positive correlation, while blue represents negative correlation. BA, Brodmann area; CAS, clinical activity score; FT3, free triiodothyronine; FT4, free thyroxine; MSD, mean squared displacement; NG, non‐Gaussianity; NGRad, radial non‐Gaussianity; ON, optic nerve; OT, optic tract; QIV, q‐space inverse variance; RTAP, return‐to‐axis probability; RTOP, return‐to‐origin probability; RTPP, return‐to‐plane probability; TAO, thyroid‐associated ophthalmopathy; TG, thyroglobulin; TGAb, thyroglobulin antibody; TPOAb, thyroid peroxidase antibody; TRAb, thyrotropin receptor antibody; TSH, thyroid stimulating hormone.

MAP‐MRI parameters of orbital and intracranial visual pathway demonstrated no significant correlations with the demographic (age, sex and TAO duration) and clinical variables (Figure 5). Although a trend of positive association was noted between the CAS score and QIV_ON, it did not reach statistical significance (*r* = 0.464, *p* = 0.027).

## 4 Discussion

This study is the first to apply MAP‐MRI in the entire visual pathway extending from orbital to intracranial for the diagnosis of DON. The main three findings were as follows: first, nearly all orbital MAP‐MRI parameters showed significant differences between DON patients and non‐DON patients, among which the QIV value exhibited the highest diagnostic efficacy. Second, as for intracranial visual pathway MAP‐MRI parameters, the NGRad value of OT, as well as the QIV and MSD values of BA17, demonstrated intergroup differences. Notably, the QIV value of BA17 achieved the highest diagnostic efficacy. Third, combining the MAP‐MRI parameters of the orbital and intracranial visual pathways could achieve the best diagnostic performance.

The MAP‐MRI quantifies tissue microstructure through a set of eight parameters derived from water diffusion displacement. Among them, the NG parameter captures the deviation of the spin displacement's probability density function from its Gaussian approximation, serving as an alternative to kurtosis metrics [16]. Its axial (NGAx) and radial (NGRad) components, representing diffusion directionality, reveal local variations in NG and reflect tissue microstructural complexity, similar to Mean Kurtosis (MK) [26]. The parameters RTOP, RTAP, and RTPP can estimate the probability that a water molecule will diffuse and return to a specific point (origin, axis, or plane) within a given time, reflecting how restricted the diffusion space is [27]. These parameters are associated with axonal integrity and myelination, and reflect overall restrictions and cellularity [18]. Concurrent reductions in these parameters suggest decreased neuronal density and increased free diffusion in tissue [28]. MSD, a measure of proton diffusion distance (whether restricted or hindered), is closely related to the classical mean diffusivity (MD) metric [29]. An increase in MSD values indicates enhanced free water diffusion resulting from diminished diffusion anisotropy due to compromised fiber integrity [30]. QIV, which is similar to MSD and defined as the inverse variance of the geometric mean signal, serves as a non‐Gaussian diffusion biomarker for pathologies that are inaccessible to conventional diffusion imaging [31]. QIV exhibits heightened sensitivity to slow or restricted diffusion compartments and tissue compositional heterogeneity [32].

Our study reported significant decrease in non‐gaussianity and return‐to‐origin probability parameters within the ON of DON patients. These findings reflected a reduction in restricted diffusion, indicating impaired microstructural barrier function and disrupted fiber integrity of the ON. It was speculated that these results may be closely associated with compressive ischemia of the ON in DON patients, which leads to inflammatory cell infiltration and myelin sheath disintegration. A previous study revealed that inflammatory‐related molecules and immune cell infiltration play a critical role in the pathogenesis of DON [33]. In our study, the increased MSD values suggested the presence of extracellular edema within the ON, a consequence of inflammatory responses, resulting in enhanced free water diffusion. Furthermore, the increase in QIV also supported microstructural disorganization and disruption within the ON in DON patients. Previous diffusion imaging studies have similarly reported reductions in directional metrics such as axial diffusivity (AD), axial kurtosis (AK), radial kurtosis (RK), MK, and increases in MD within the optic nerve of DON patients [4, 14, 34]. These findings were consistent with ours, collectively indicating compromised optic nerve fiber integrity in DON patients and reduced diffusion restrictions.

Besides, extensive microstructural abnormalities on the intracranial visual pathway were discovered in our study. Trans‐synaptic degeneration is proposed as a potential pathological mechanism underlying these changes [4]. BA17, a critical hub for primary visual processing, receives direct input from the lateral geniculate nucleus (LGN) and transmits information to higher‐order visual centers. Hu et al. reported decreased kurtosis fractional anisotropy (KFA) values in the visual cortex of DON patients, suggesting cell dissolution, fiber disruption, or tissue loss in the visual cortex [13]. Consistently, our findings on MSD values of BA17 reflected increased extracellular space expansion and enhanced diffusion freedom in the visual cortex, which may result from decreased cellular density and structural disintegration. Concurrently, the increased QIV values indicated enhanced microstructural heterogeneity in BA17. These findings may be associated with the disintegration and impairment of visual cortical cells, as well as structural abnormalities induced by necrosis, fibrosis, and other factors. Microstructural disruption in BA17 could lead to dysfunctions in the initial processing and subsequent transmission of visual information. Therefore, it is hypothesized that changes in the MAP‐MRI parameters of the visual cortex in DON patients may be linked to local microstructural damage and visual processing disorders.

There were no correlations between clinical characteristics and MAP‐MRI parameters. Although CAS differed between DON and non‐DON groups, it showed no significant correlation with the MAP‐MRI parameters. The CAS is primarily designed to assess inflammatory signs in the anterior orbital segment, such as redness, swelling, and pain. It has limited direct correlation with the pathological processes affecting the posterior optic nerve and intracranial visual pathways. Besides, anterior orbital involvement is typically more severe in DON patients. Therefore, a higher CAS is a normal clinical manifestation in this cohort. This observation was consistent with the baseline characteristics of the study population described by Hu et al. [13]. This indicated that the diagnostic utility of the MAP‐MRI model for DON is independent of thyroid function indicators and CAS activity.

The integration of MAP‐MRI parameters from both the orbital ON and the intracranial visual pathway yielded a diagnostic model for DON with superior performance and higher sensitivity compared to models based on either dataset alone. By capturing information on diffusion restriction, displacement, and tissue complexity through its eight parameters, MAP‐MRI provides a more comprehensive characterization of tissue microstructure. Comprehensive analysis of MAP‐MRI parameters along the entire visual pathway elucidates disease extent, providing novel insights for the early diagnosis and optimized management of DON. This diagnostic model facilitates early detection and helps minimize diagnostic oversights.

Our study has several limitations. First, the small sample size, a consequence of the low incidence of DON, and the lack of external validation may limit the generalizability of our findings, necessitating future large‐scale studies. Second, as a cross‐sectional analysis, it cannot track disease progression. Longitudinal studies are needed to investigate the entire course of DON. Finally, future research would benefit from multi‐modal MRI analysis to develop a more integrative pathophysiological model of the disease.

In conclusion, the comprehensive profiling of MAP‐MRI parameters along the entire visual pathway enables a novel model for diagnosing DON. This model improved diagnostic performance, providing certain support for the early diagnosis and accurate treatment for DON patients.

## Funding

This study has received funding by the National Natural Science Foundation of China (no: 82471127).

## Ethics Statement

This study was approved by the ethics review committee of the first affiliated hospital of sun yat‐sen university (NO: [2019]061).

## Consent

Written informed consent was obtained from all subjects (patients) in this study.

## Conflicts of Interest

The authors declare no conflicts of interest.

## Supporting information


**Table S1:** Comparison of MAP parameters of optic nerve and visual pathway among DON, non‐DON and HC groups.

## Data Availability

The data that support the findings of this study are available from the corresponding author upon reasonable request.
