# Supplementary material for: The Diagnostic Value of Mean Apparent Propagator‐MRI in Dysthyroid Optic Neuropathy: A Quantitative Analysis of the Entire Visual Pathway
Source: CNS Neurosci Ther. 2026 Feb 11;32(2):e70793. doi: 10.1002/cns.70793 (PMC12894417; doi:10.1002/cns.70793)
Supplement: Supplementary file 1 — Table S1: Comparison of MAP parameters of optic nerve and visual pathway among DON, non‐DON and HC groups. [file CNS-32-e70793-s001.docx]

Supplementary Table 1 Comparison of MAP parameters of optic nerve and visual pathway among DON, non-DON and HC groups.

|  | |  | | Three group comparison | | Post-hoc analysis | | |
| --- | --- | --- | --- | --- | --- | --- | --- | --- |
|  | DON | non-DON | HC | *p* | DON vs non-DON | | DON vs HC | non-DON vs HC |
| Orbital-MAP |  |  |  |  |  | |  |  |
| NG_ON | 0.34±0.03 | 0.35±0.02 | 0.37±0.03 | <0.001^a^ | 0.005^b^ | | <0.001 | 0.005 |
| NGAx_ON | 0.28(0.27, 0.29) | 0.29(0.27, 0.30) | 0.299(0.29, 0.31) | <0.001^a^ | 0.389 | | <0.001 | 0.001 |
| NGRad_ON | 0.176±0.019 | 0.193±0.021 | 0.189±0.022 | <0.001^a^ | <0.001^b^ | | 0.004 | 0.769 |
| QIV_ON | 16.33(13.83, 19.56) | 11.57(9.67, 13.72) | 13.50(11.06, 15.04) | <0.001^a^ | 0.001^b^ | | <0.001 | 0.315 |
| MSD_ON | 19.32(17.89, 20.86) | 17.30(15.34, 18.66) | 19.47(17.24, 20.47) | <0.001^a^ | <0.001^b^ | | 0.397 | 0.004 |
| RTAP_ON | 7.64(6.62, 8.74) | 9.24(8.12, 11.28) | 7.89(6.98, 9.47) | <0.001^a^ | <0.001^b^ | | 0.767 | 0.013 |
| RTOP_ON | 5.25(4.43, 6.57) | 7.36(5.77, 9.72) | 5.69(4.86, 7.69) | <0.001^a^ | <0.001^b^ | | 0.242 | 0.024 |
| RTPP_ON | 4.98(4.75, 5.35) | 5.58(5.18, 5.83) | 5.28(5.02, 5.82) | <0.001^a^ | <0.001^b^ | | 0.002 | 0.672 |
| Visual pathway-MAP |  |  |  |  |  | |  |  |
| NG_OT | 0.29±0.02 | 0.27±0.03 | 0.27±0.02 | 0.009^a^ | 0.060 | | 0.012 | 0.860 |
| NGAx_OT | 0.24(0.23, 0.26) | 0.23(0.22, 0.25) | 0.23(0.21, 0.24) | 0.060 | 0.055 | | 0.032 | 0.823 |
| NGRad_OT | 0.15±0.02 | 0.11±0.02 | 0.14±0.01 | 0.001^a^ | 0.028^b^ | | 0.001 | 0.653 |
| QIV_OT | 25.33±6.11 | 24.12±2.92 | 23.76±2.91 | 0.455 | 0.714 | | 0.510 | 0.953 |
| MSD_OT | 25.05±2.53 | 24.03±2.68 | 24.21±2.02 | 0.233 | 0.348 | | 0.540 | 0.960 |
| RTAP_OT | 4.55±0.58 | 4.49±0.62 | 4.46±0.45 | 0.819 | 0.930 | | 0.805 | 0.967 |
| RTOP_OT | 3.26(2.81, 3.76) | 2.98(2.63, 3.23) | 2.88(2.62, 3.50) | 0.347 | 0.250 | | 0.183 | 0.873 |
| RTPP_OT | 4.93±0.32 | 4.90±0.28 | 4.89±0.21 | 0.815 | 0.866 | | 0.829 | 0.998 |
| NG_OR | 0.25±0.01 | 0.25±0.01 | 0.24±0.01 | 0.176 | 0.408 | | 0.125 | 0.914 |
| NGAx_OR | 0.21(0.20, 0.22) | 0.21(0.21, 0.21) | 0.21(0.21, 0.21) | 0.508 | 0.576 | | 0.280 | 0.462 |
| NGRad_OR | 0.13(0.12, 0.13) | 0.13(0.12, 0.13) | 0.13(0.12, 0.13 | 0.385 | 0.213 | | 0.267 | 0.684 |
| QIV_OR | 19.75(18.86, 21.01) | 20.39(18.97, 22.40) | 19.36(18.80, 20.07) | 0.299 | 0.761 | | 0.225 | 0.155 |
| MSD_OR | 15.52(15.10, 15.95) | 15.43(15.02, 16.17) | 15.06(14.81, 15.23) | 0.038^a^ | 0.717 | | 0.050 | 0.148 |
| RTAP_OR | 6.95(6.67, 7.18) | 6.91(6.60, 7.26) | 7.06(6.97, 7.17) | 0.303 | 0.643 | | 0.225 | 0.169 |
| RTOP_OR | 4.69(4.42, 4.89) | 4.63(4.39, 4.89) | 4.76(4.68, 4.84) | 0.413 | 0.632 | | 0.460 | 0.169 |
| RTPP_OR | 5.45(5.39, 5.55) | 5.45(5.41, 5.53) | 5.49(5.43, 5.54) | 0.387 | 0.835 | | 0.399 | 0.141 |
| NG_BA17 | 0.18(0.17, 0.19) | 0.17(0.15, 0.18) | 0.17(0.15, 0.18) | 0.006^a^ | 0.172 | | 0.004 | 0.697 |
| NGAx_BA17 | 0.15(0.14, 0.16) | 0.14(0.13, 0.15) | 0.14(0.13, 0.15) | 0.008^a^ | 0.191 | | 0.006 | 0.770 |
| NGRad_BA17 | 0.10(0.09, 0.11) | 0.10(0.08, 0.10) | 0.09(0.08, 0.10) | 0.004^a^ | 0.080 | | 0.003 | 0.993 |
| QIV_BA17 | 27.79±5.28 | 24.31±4.12 | 23.24±3.24 | <0.001^a^ | 0.009^b^ | | <0.001 | 0.621 |
| MSD_ BA17 | 16.10±2.31 | 14.80±1.87 | 14.30±1.69 | 0.002^a^ | 0.045^b^ | | 0.002 | 0.618 |
| RTAP_ BA17 | 4.22(4.00, 4.55) | 4.35(3.78, 4.61) | 4.13(3.84, 4.41) | 0.366 | 0.848 | | 0.225 | 0.225 |
| RTOP_ BA17 | 3.46(3.02, 3.88) | 3.45(2.67, 3.77) | 3.12(2.90, 3.61) | 0.534 | 0.835 | | 0.243 | 0.502 |
| RTPP_ BA17 | 5.10(4.91, 5.28) | 5.10(4.73, 5.29) | 4.95(4.56, 5.15) | 0.191 | 0.620 | | 0.062 | 0.284 |
| NG_BA18 | 0.19(0.17, 0.19) | 0.17(0.15, 0.18) | 0.17(0.16, 0.18) | 0.008^a^ | 0.140 | | 0.007 | 0.335 |
| NGAx_BA18 | 0.15(0.14, 0.16) | 0.14(0.12, 0.15) | 0.14(0.13, 0.15) | 0.009^a^ | 0.139 | | 0.008 | 0.343 |
| NGRad_BA18 | 0.10(0.09, 0.11) | 0.10(0.08, 0.10) | 0.09(0.09, 0.10) | 0.010^a^ | 0.156 | | 0.009 | 0.339 |
| QIV_BA18 | 23.50(20.53, 26.19 | 21.35(19.78, 23.43) | 21.65 (20.29, 22.94) | 0.086 | 0.084 | | 0.040 | 0.632 |
| MSD_ BA18 | 14.96±2.10 | 13.93±1.55 | 14.06±1.34 | 0.046^a^ | 0.074 | | 0.128 | 0.957 |
| RTAP_ BA18 | 4.55(4.08, 4.90) | 4.62(3.70, 4.73) | 4.18(3.81, 4.33) | 0.016^a^ | 0.520 | | 0.018 | 0.125 |
| RTOP_ BA18 | 3.81(3.35, 4.35) | 3.99(2.77, 4.37) | 3.27(2.90, 3.70) | 0.027^a^ | 0.654 | | 0.034 | 0.130 |
| RTPP_ BA18 | 5.05(4.86, 5.35) | 4.99(4.49, 5.15) | 4.84(4.65, 5.05) | 0.081 | 0.146 | | 0.027 | 0.554 |
| NG_BA19 | 0.19±0.02 | 0.18±0.01 | 0.18±0.01 | 0.016 ^a^ | 0.764 | | 0.013 | 0.272 |
| NGAx_BA19 | 0.15±0.01 | 0.15±0.01 | 0.15±0.01 | 0.021 ^a^ | 0.903 | | 0.018 | 0.275 |
| NGRad_BA19 | 0.10±0.01 | 0.10±0.01 | 0.10±0.01 | 0.016 ^a^ | 0.778 | | 0.013 | 0.274 |
| QIV_BA19 | 24.96(23.35, 28.21) | 23.73(22.60, 26.99) | 23.27(21.89, 24.92) | 0.192 | 0.397 | | 0.074 | 0.338 |
| MSD_ BA19 | 15.31±1.44 | 14.97±1.48 | 14.70±1.15 | 0.221 | 0.619 | | 0.192 | 0.724 |
| RTAP_ BA19 | 4.59(4.47, 4.88) | 4.67(4.48, 4.93) | 4.54(4.23, 4.68) | 0.107 | 0.609 | | 0.135 | 0.041 |
| RTOP_ BA19 | 4.01(3.57, 4.34) | 3.96(3.81, 4.33) | 3.87(3.33, 4.07) | 0.134 | 0.609 | | 0.121 | 0.068 |
| RTPP_ BA19 | 5.15±0.27 | 5.14±0.22 | 5.03±0.24 | 0.113 | 0.990 | | 0.178 | 0.264 |

The numeric data are reported as the mean±standard deviation or the median with an interquartile range (25%, 75%).

a Statistical significance is indicated by *p*<0.05 when comprision among TAO with and without DON, and healthy controls were done.

b Statistical significance is indicated by *p*<0.05 when comprision among TAO with and without DON were done.

Abbreviation: MAP mean apparent propagator, TAO thyroid-associated ophthalmopathy, DON dysthyroid optic neuropathy, HC healthy controls, ON optic nerve, OT optic tract, OR optic radiation, NG non-Gaussianity, NGAx axial non-Gaussianity, NGRad radial non-Gaussianity, QIV q-space inverse variance, MSD mean squared displacement, RTAP return-to-axis probability, RTOP return-to-origin probability, RTPP return-to-plane probability, BA Brodmann area.
